# The WHOQOL-BREF: a modern psychometric evaluation of its internal construct validity in people with multiple sclerosis

**DOI:** 10.1007/s11136-020-02463-z

**Published:** 2020-03-19

**Authors:** I. M. Pomeroy, A. Tennant, R. J. Mills, TONiC Study Group, C. A. Young

**Affiliations:** 1 Department of Neurology, Walton Centre NHS Foundation Trust, Liverpool, UK; 2 Leeds Institute of Rheumatic and Musculoskeletal Medicine, University of Leeds, Leeds, UK; 3 University of Liverpool, Liverpool, UK

**Keywords:** Multiple sclerosis, Rasch analysis, Clinical outcomes, Quality of life

## Abstract

**Purpose:**

Symptoms of Multiple Sclerosis (MS) differentially impact upon quality of life (QoL) and a comprehensive measure is required for use in observational and interventional studies. This study examines the abbreviated World Health Organisation Quality of Life tool (WHOQOL-BREF) which was designed to be used as a broad measure of QoL across different cultures and diseases.

**Methods:**

Data were collected from 3186 subjects as part of the TONiC study in MS and was examined with a systematic, iterative approach using Rasch analysis to investigate the internal construct validity of the WHOQOL-BREF.

**Results:**

Mean age was 49.8 years (SD 11.8), disease duration was 11.2 years (SD 9.6) and 73.2% were female. Subjects represented all stages of MS with EDSS scores of 0–4, 4.5–6.5, 7–7.5 and ≥ 8 seen in 49.8%, 38.5%, 6.8% and 4.9% of patients, respectively. Using a super-item approach, it was possible to demonstrate fit to the assumptions of the Rasch model for 3 of the 4 domains of the WHOQOL-BREF (physical, psychological and environment) as well as a broad 24-item total score. In addition, item subsets derived from the stem of each question were shown to function as novel scales measuring impact and life satisfaction. We have provided transformation tables from ordinal raw scores to interval scales where data are complete.

**Conclusions:**

The validation of multiple conceptual frameworks validates the WHOQOL-BREF as a powerful and flexible end-point for use in clinical trials and in testing conceptual models of factors influencing QoL in MS.

## Introduction

Multiple Sclerosis (MS), is a neurodegenerative disorder that affects all aspects of life [1–3]. Fluctuating symptoms and a variable decline in functioning, can differentially impact upon perceived quality of life [4]⁠. As all aspects of life can be affected, a conceptual model that draws on the biomedical and social sciences paradigm and which incorporates, biological, psychological and social aspects of health is needed to represent that variable experience [5]⁠. One model was postulated by Wilson and Cleary in 1995 which incorporates aspects of health status (e.g. symptoms and functioning) and separately, QoL, as well as potential environmental moderators and psychological mediators [6]⁠. The model is consistent with recently identified priority outcomes for trials of disease modifying therapies in MS, that is symptoms, disability (functioning) and QoL [7]⁠.

Given this, one task in order to operationalize the model is to select the Patient Reported Outcome Measures (PROM’s), or other assessments that should be used to specify the model. There is no clear consensus on a scale to measure QoL in MS, for example five recent phase 3 treatment trials since 2012 have employed a variety of scales including the Short Form Survey (SF-36), EQ5D, MSQOL-54, and Functional Assessment of Multiple Sclerosis (FAMS) [8–12]⁠. The SF-36 and EQ5D are fundamentally measures of health status with functioning as a predominant construct [13]⁠, whereas, the Wilson and Cleary model differentiates between functioning and QoL. The MSQOL-54 is a derivative of the SF36 expanded to include items relevant for people with MS and as such is thought to be a end-point measure, as is the FAMS which combines physical and social functioning as well as well-being.

The current study uses data from a large population of MS patients recruited into the TONiC study https://tonic.thewaltoncentre.nhs.uk/ to evaluate the abbreviated World Health Organisation Quality of Life tool (WHOQOL-BREF) as a potential QoL outcome for such a conceptual model. This 26-item scale was developed across 18 countries to be used as a broad measure of QoL across different cultures and diseases [14]. Structural equational modelling of the original scale showed that the items could be drawn together into 4 domains; physical health, psychological, social relationships and environment. As well as evaluating the conventional domain-based scoring of the WHOQOL-BREF, this paper suggests a different way of summating the item set to provide just two distinct domains for consideration, as well as an overall score.

## Methods

### Recruitment

Patients were recruited as part of the Trajectories of Outcomes in Neurological Conditions (TONiC) study investigating predictors of quality of life in chronic neurological disease. Subjects with MS were recruited from 23 centres across the UK and were asked to complete a questionnaire pack which included the WHOQOL-BREF amongst a variety of PROM’s designed to map on to the International Classification of Functioning, Disability and Health (ICF) Brief Core Set for MS [15]. The project aimed to recruit subjects across a broad range of ages, diseases subtypes and disabilities. Patients who were physically unable to complete the questionnaires were permitted to use a scribe to report their answers on the form. All participants received written information and informed consent was obtained from all individual participants included in the study prior to enrolment.

### Rasch analysis

The Rasch model provides a systematic and objective method to evaluate a set of criteria consistent with fundamental measurement, that is the type of measurement normally found in the physical sciences [16]⁠. These criteria include the stochastic (probabilistic) ordering of items, monotonicity (increase in item responses consistent with underlying trait), local item independence (zero correlation between items when conditioned on the score), unidimensionality and group invariance (no difference in response to item by group membership when at the same level of (in this case) QoL [17]. Thus data from a scale are tested against the requirements of the Rasch model in a process widely known as Rasch analysis. Full details of this process are given elsewhere [18].

⁠Recent methodological developments have updated the previously published guidelines. Thus, local item independence was examined by constructing a residual item correlation matrix between all items. The residual of an item is the difference between the estimate of item difficulty given the model, and the observed item difficulty, standardised to a mean of zero and standard deviation of 1. Residual correlations of + 0.2 above the average correlation are considered an indicator of a breach of the local independence criteria [19]⁠. That is, there is something else, other than the trait being measured (e.g. QoL) that is causing an association between the items. This may be due to the fact that the items are close replications of one another, or multidimensionality is present. When local item dependency is observed, it is accommodated through a strategy of grouping items, either along the lines of a priori known domains where the grouping is consistent with a testlet, or from other evidence where the grouping is post hoc, contingent on the analysis, and referred to as ‘super items’ [20, 21]. This latter approach can include two super items, created by allocating alternative items to each super item, on the basis that as the scale (domain) was supposed to be summated into a single score, then alternative items should demonstrate a near perfect latent correlation between the two, while absorbing most, if not all, local dependency within the item set. Analysis using two super items has the advantage of providing a more robust conditional chi-square test of fit, together with the proportion of variance retained in a bi-factor equivalent solution, consistent with the explained common variance (EVC) in the bi-factor literature [22–24]⁠. A bi-factor solution is where the latent estimate is based upon the first (Rasch) factor, upon which all items load but also load onto secondary factors EVC should be > 0.9 if the scale is to be considered essentially unidimensional, that is greater than 90% of the variance is common and retained in the latent estimate.

The data were further evaluated for differential item functioning (DIF) by age group, gender, MS subtype and duration [25]⁠. Analysis of DIF examines whether each item performs equally across different subgroups, given the same level of the underlying trait. So, contextual characteristics who have the same level of quality of life. Evidence of DIF was sought from graphical displays of group-specific item characteristic curves, and statistically if the *p*-value derived from an ANOVA analysis was significant at the 5% level with a Bonferroni correction applied. Where a testlet or super-item solution is obtained, and DIF is shown to be still present, the substantive nature of this DIF is tested by comparing unadjusted and adjusted person estimates. Should the *t* test of this comparison (for paired or repeated measures) be significant, then an effect size of the difference is calculated which should be less than 0.1, in which case DIF is deemed to be small and no action is taken [26]. All analyses were performed using RUMM2030 software [27].

Targeting of each scale was assessed by person-threshold (transition between categories) distribution plots, which plot the range of QoL recorded by subjects against the range measured by each scale. Spearman’s Rho correlation coefficients were calculated to ascertain concurrent validity between the derived scales and the established scales included in the TONiC study: the EQ5D and Leeds MS QoL (LMSQoL) [28, 29]. Finally, the precision of the various domains of the WHOQOL-BREF was examined with respect to the standard error of measurement (SEM) and the smallest detectable difference (SDD) [30, 31]⁠.

Thus, the properties of the WHOQOL-BREF were first examined according to the original structure of physical, psychological, environment and social health subscales, then two new domains were considered reflecting the stems of the items, that is ‘impact’ and ‘satisfaction’ and finally, the data were examined for a 24-item total solution. Where a solution was identified to fit the assumptions of the Rasch model, a conversion table was calculated to enable transformation of the original ordinal scores to an interval scale without the use of specialist software.

## Results

### Subjects

3186 people with MS were recruited into the study by mid-2017 and had returned the baseline questionnaire. Mean age was 49.8 years (SD 11.8) and mean duration of MS (since diagnosis) was 11.2 years (SD9.6). Almost three quarters (73.2%) were female. Three fifths (60.3%) had a Relapsing Remitting form of MS (RRMS), 11.7% Primary Progressive; 22.9% Secondary Progressive, and 5.1% a rapidly evolving form of RRMS. Almost half (49.8%) had an EDSS of 0–4.0; 38.5% were at level 4.5–6.5; 6.75% at level 7–7.5 and 4.9% had an EDSS ≥ 8.0.

### Rasch analysis

Data from the WHOQOL-BREF were fit to the Rasch model in a series of analyses representing different grouping of items shown in Table 1, ideal values for fit statistics derived from Rasch analysis literature are provided within the table [19, 32]. The requirement for local independence of items was found to be breached for all groups. For example, in the 24-item-based scale, the items ‘How much do you enjoy life’ and ‘To what extent do you feel your life to be meaningful’, had a residual correlation of 0.481. For the physical subscale, whose average residual correlation was − 0.16, the items ‘How well are you able to get around’ and ‘How satisfied are you with your ability to perform your daily activities’ had a residual correlation of 0.198. As a consequence, in practice all the item groups (domains) were resolved into two super items.

**Table 1:** Fit of WHOQOL-BREF domains to Rasch model

Analysis	Scale	Standard deviation of residuals		Chi-square fit statistic		Reliability		Unidimen- sionality	Super item/testlet analysis		
		Items	Persons	Value and (d*f*)	*P*	PSI	Alpha	*t* test; % < 5% (LCI)	Latent correlation	Explained common variance	Conditional test of fit Chi-square *P* value
1	Physical—7-item	9.76	1.23	1013.8 (63)	< 0.001	0.84	0.84	10.17	–	–	
2	Physical—2 super items	2.81	0.95	32.6 (18)	0.19	0.85	0.84	3.27	1.0	1.0	0.321
3	Psychological—6 item	6.89	1.23	336.0 (54)	< 0.001	0.84	0.85	6.46 (5.7)	–	–	–
4	Psychological—2 super items	0.34	0.88	8.9 (18)	0.96	0.88	0.89	4.65	1.0	1.0	0.041
5	Social—3 items	4.90	1.09	190.6 (21)	< 0.001	0.61	0.68	0.04	–	–	–
6	Environment—8 items	3.04	1.20	202.6 (72)	< 0.001	0.83	0.84	7.85 (7.1)	–	–	–
7	Environment—2 super items	0.63	0.83	33.2 (18)	0.016	0.80	0.83	3.36	0.91	0.96	0.061
8	Impact domain	6.23	1.43	1014.8 (126)	< 0.001	0.90	0.89	11.68 (10.9)	–	–	–
9	2 Super items	0.86	0.93	14.5 (18)	0.696	0.92	0.93	4.22	1.0	1.0	0.199
10	Life satisfaction domain	5.82	1.26	640.7 (90)	< 0.001	0.84	0.84	10.19 (9.4)	–	–	–
11	Life satisfaction 2-super items	0.92	0.94	15.4 (18)	0.637	0.88		4.55	1.0	1.0	0.235
12	24 items	7.46	1.59	2381.6 (216)	< 0.001	0.93	0.93	17.32	–	–	–
13	4 domain-based testlets	5.89	1.08	96.8 (36)	< 0.001	0.86	0.84	5.01 (4.2)	0.83	0.92	–
14	2 Super items	0.09	0.99	11.45 (18)	0.874	0.94	0.95	4.59	1.0	1.0	0.407
	Ideal values	**1.0**	**1.0**		** > 0.01**	** > 0.7**	** > 0.7**	** < 5.0**	** > 0.9**	** > 0.9**	** > 0.01**

### Physical health

The 7-item physical subscale (Table 1, Analysis 1) showed significant misfit to the model, including a breach of the local independence solution, multidimensionality, and substantial DIF by age, duration and disease subtype.

**Fig. 1:**
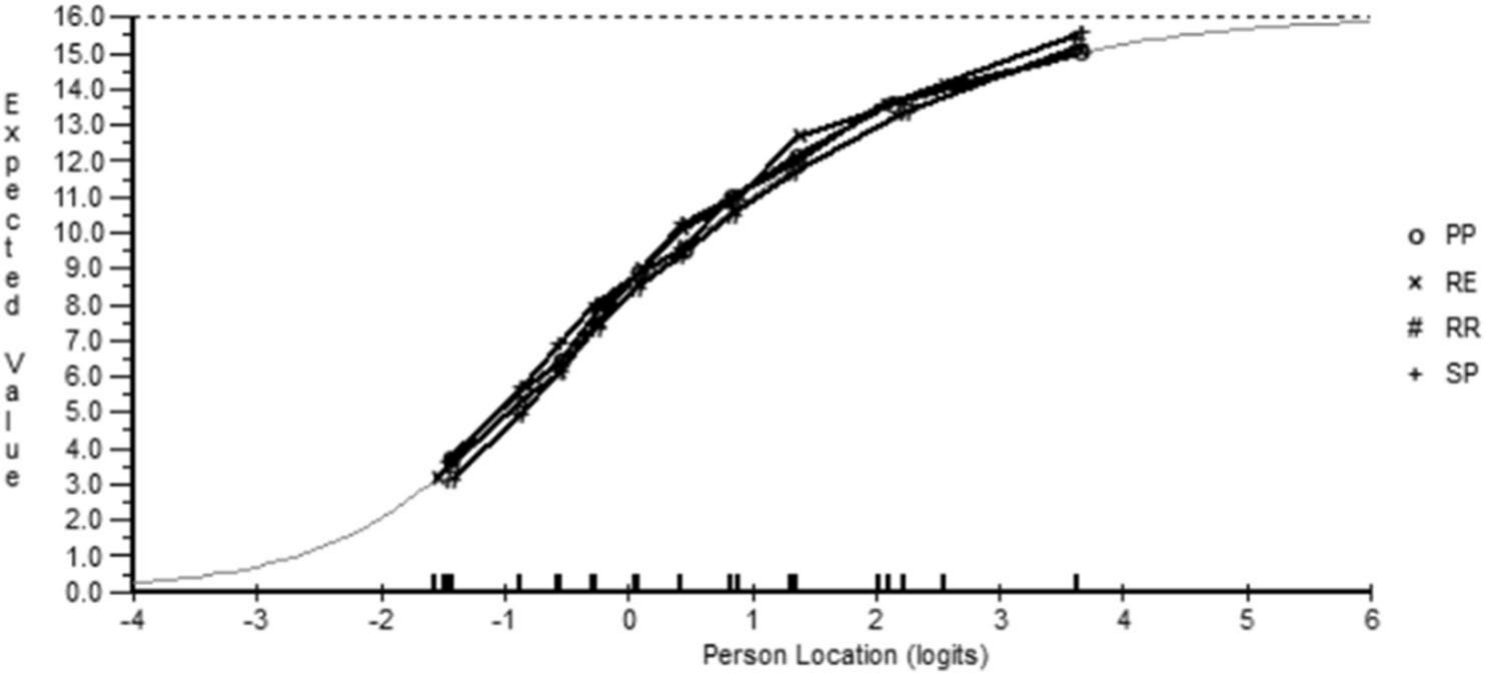
Physical health—differential item functioning by disease subtype. DIF plot showing slight deviation for rapidly evolving (RE) RRMS in the physical health domain

All thresholds were ordered, supporting the monotonicity requirement. The two ‘super-item’ approach resolved the fit, with the two latent estimates perfectly correlated, and the explained common variance at 1.0, indicating that no unique variance had been discarded to achieve a unidimensional latent estimate which was confirmed by the *t* test (Analysis 2). However, DIF remained. Graphically it was hard to determine where the DIF was present, but the MS subtype showed some slight deviation for rapidly evolving (RE) RRMS (Fig. 1). Substantive DIF was thus tested by contrasting the estimates from a split solution (RE + the rest) against an unsplit solution, anchored by the split super-item parameters. The effect size of such a contrast was 0.032. As such no further action was taken for DIF on the physical subscale.

### Psychological health

The 6-item psychological scale also showed misfit to the model, with local dependencies, multidimensionality and significant DIF (Analysis 3). All item thresholds were ordered. The two super items showed good fit to the model (Analysis 4). While duration was invariant, DIF was present for age, gender and MS subtype. Graphical interpretation was not helpful, but it appeared that females showed the most deviation from the expected curve, and so a super item was split by gender. The effect size for the difference between estimates was 0.031, no further action was taken for DIF and the original unsplit estimate used.

### Social relationships

The three-item social relationships scale (Analysis 5) failed to show fit to the Rasch model. With just three items, the test for unidimensionality was underpowered. The reliability of the scale remained below that considered a minimum for group use. As such, no resolution was attempted.

### Environment

The original 8 item environment scale (Analysis 6) showed misfit to the model, multidimensionality and DIF by age and disease subtype. The two super items demonstrated adequate fit within a bi-factor equivalent solution, having shed some 4% of the variance to achieve a unidimensional latent estimate (Analysis 7). DIF was absent for all items.

### Impact and satisfaction scales

It is also possible to view the 24-item set from a different perspective, based upon the format of the stem of each question. In this way two separate scales assessing impact (items 3–15 and 26) and satisfaction (items 16–25) may be considered. The 14-item impact scale (Analysis 8) did not fit the Rasch model. While all thresholds were ordered, the scale was multidimensional, DIF manifested for all contextual factors, and local item dependencies were present. A two super-item approach resolved the issue (Analysis 9). While DIF was present for age and disease subtype, no variation was observed from the graphical interpretation.

**Fig. 2:**
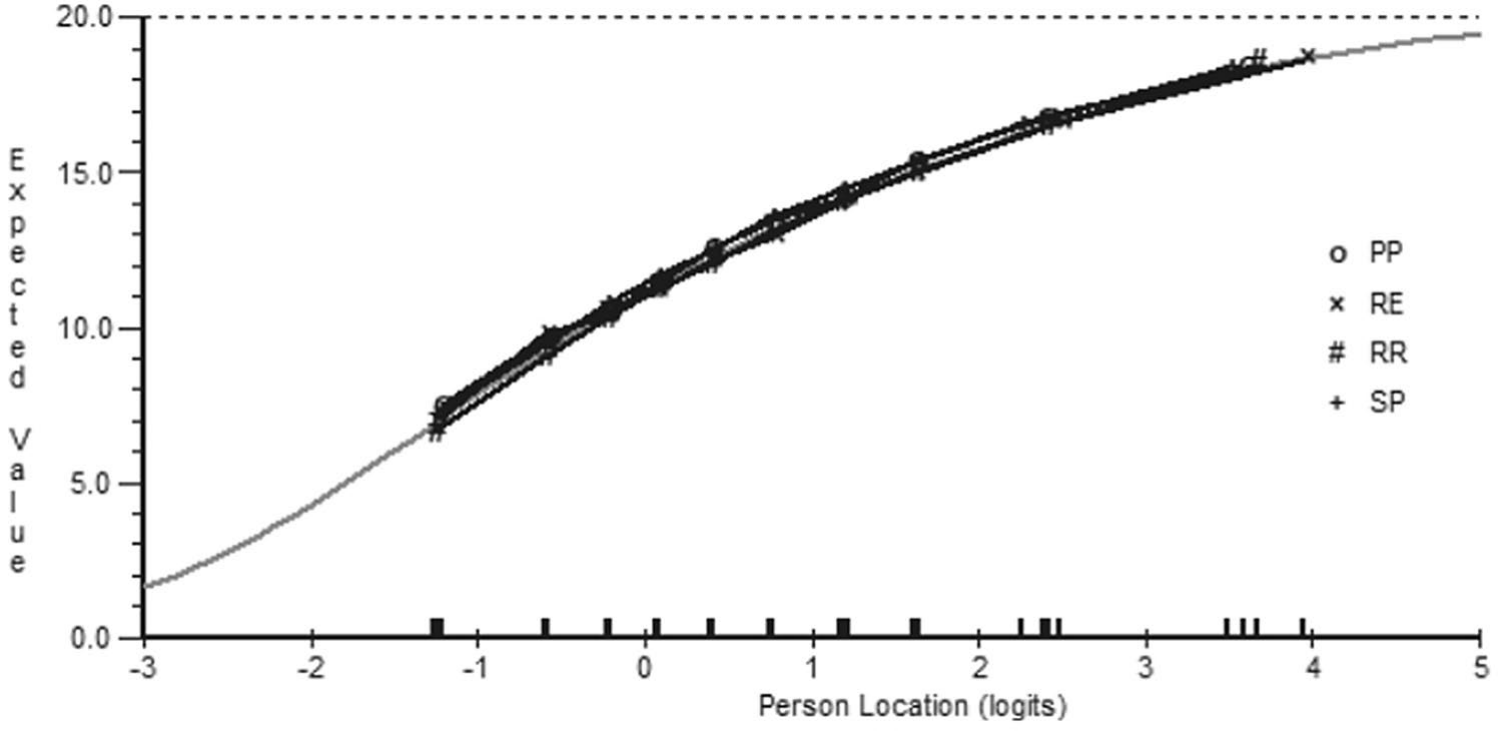
Differential item functioning (DIF) by disease subtype on one super item in the life satisfaction domain. DIF plot displaying marginal DIF for one super item in the life satisfaction domain

**Fig. 3:**
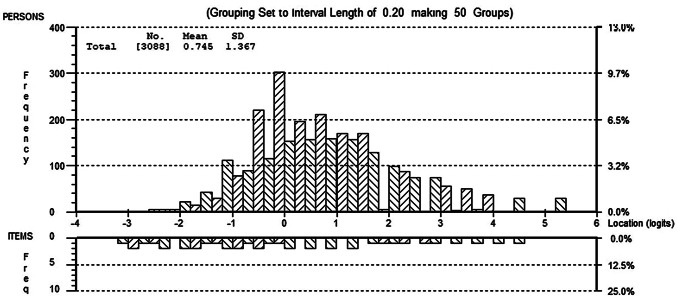
Person-item distribution of the life satisfaction domain. Person-item distribution plot showing well matched distributions of levels of life satisfaction between the scale items and the study population

A similar result as found for the 10-item satisfaction scale. Initially the data did not fit the model and showed evidence of multidimensionality, local item dependencies, two disordered thresholds and DIF on all contextual factors. (Analysis 10). Combining items into two super items resulted in satisfactory fit and unidimensionality, having discarded none of the variance (Analysis 11). DIF was still in evidence for age and disease subtype, although it was almost impossible to visualize any difference in the group-specific item characteristic curves (e.g. see Fig. 2) and, given earlier findings and their magnitude of effect size observed, no further action was taken. The targeting of the satisfaction domain was good, showing a slightly higher level of satisfaction than the average of the scale, which itself showed a near perfect distribution across the trait (Fig. 3).

### 24 item total scale

The 24-item scale showed considerable misfit, multidimensionality, local dependencies, and DIF across all contextual factors, often for several items (Analysis 12). Two strategies were considered to see if a total score was valid: a testlet strategy based upon the four domains, and a strategy based upon alternative items allocated to two super items.

Creating 4 testlets according the 4 original subscales failed to resolve fit to the model (Analysis 13). The psychological and social testlets were dependent, DIF was present for all contextual factors, and 8% of the variance had to be discarded in order to achieve a unidimensional latent estimate (although adequate under a bi-factor solution).

In contrast, the two super-item analysis displayed near perfect fit, with excellent reliability, and none of the variance was discarded to obtain the unidimensional latent estimate (Analysis 14). DIF remained on all but the duration factor, but here the sample size was influencing small group differences that were not substantive. For example, splitting the youngest age off in one super item, and comparing unsplit with (Anchored) split age gave an effect size for the difference in person estimates of 0.05.

Transformations of the ordinal raw score of the above scales to interval scale latent metrics are available in Table 2.

**Table 2:** Raw score to interval-scale transformation of domains

Raw score	24 Item	Physical	Psychological	Environment	Impact	Life satisfaction
0	0.0	0.0	0.0	0.0	0.0	0.0
1	4.7	2.3	1.6	2.5	3.4	2.6
2	7.8	3.9	2.8	4.2	5.8	4.3
3	9.7	5.0	3.7	5.3	7.6	5.5
4	11.2	5.8	4.5	6.2	9.1	6.4
5	12.5	6.5	5.2	6.9	10.4	7.2
6	13.5	7.2	6.0	7.5	11.5	7.9
7	14.5	7.8	6.7	8.1	12.5	8.6
8	15.4	8.4	7.4	8.7	13.5	9.2
9	16.2	8.9	8.2	9.3	14.4	9.8
10	17.0	9.5	9.0	9.9	15.3	10.4
11	17.8	10.0	9.8	10.5	16.1	11.0
12	18.6	10.5	10.6	11.1	16.9	11.5
13	19.3	11.1	11.4	11.7	17.6	12.1
14	20.0	11.6	12.3	12.4	18.4	12.7
15	20.8	12.2	13.2	13.1	19.1	13.2
16	21.5	12.8	14.1	13.8	19.8	13.8
17	22.2	13.4	15.1	14.5	20.4	14.4
18	22.8	14.1	16.1	15.2	21.1	15.0
19	23.5	14.8	17.2	16.0	21.7	15.5
20	24.2	15.6	18.2	16.8	22.3	16.2
21	24.8	16.4	19.4	17.6	22.9	16.8
22	25.5	17.4	20.6	18.4	23.5	17.4
23	26.1	18.4	22.1	19.3	24.1	18.1
24	26.8	19.6	24.0	20.2	24.7	18.8
25	27.4	20.9		21.1	25.2	19.6
26	28.0	22.6		22.1	25.8	20.4
27	28.6	24.9		23.1	26.4	21.2
28	29.2	28.0		24.2	27.0	22.1
29	29.8			25.4	27.5	23.0
30	30.4			26.9	28.1	23.9
31	31.0			29.0	28.7	25.0
32	31.6			32.0	29.3	26.0
33	32.2				29.9	27.2
34	32.8				30.5	28.4
35	33.4				31.2	29.6
36	33.9				31.8	31.0
37	34.5				32.5	32.5
38	35.1				33.2	34.3
39	35.7				34.0	36.7
40	36.3				34.7	40.0
41	36.9				35.5	
42	37.5				36.3	
43	38.0				37.2	
44	38.6				38.1	
45	39.2				39.0	
46	39.8				40.0	
47	40.5				41.0	
48	41.1				42.1	
49	41.7				43.2	
50	42.3				44.4	
51	42.9				45.6	
52	43.6				47.0	
53	44.2				48.4	
54	44.9				50.2	
55	45.6				52.7	
56	46.2				56.0	
57	46.9					
58	47.6					
59	48.3					
60	49.1					
61	49.8					
62	50.5					
63	51.3					
64	52.1					
65	52.9					
66	53.7					
67	54.5					
68	55.3					
69	56.2					
70	57.1					
71	57.9					
72	58.9					
73	59.8					
74	60.7					
75	61.7					
76	62.7					
77	63.7					
78	64.7					
79	65.8					
80	66.9					
81	68.0					
82	69.1					
83	70.2					
84	71.4					
85	72.6					
86	73.9					
87	75.1					
88	76.4					
89	77.8					
90	79.2					
91	80.7					
92	82.4					
93	84.4					
94	87.0					
95	90.6					
96	96.0					

### Scale precision

The SEM and SDD of each domain are shown in Table 3. The original domains (excluding social which has insufficient reliability), require a difference between two groups/times of the order of 16–18% of their operational ranges to overcome the error, whereas, in descending order, the satisfaction, impact and total (24-item) domains require between 14 and 8% of their operational ranges to be above error.

**Table 3:** Precision of the WHOQOL-BREF domains and other PROMS

Domain	Mean	SD	SEM	SDC	%SDC
WHOQoL-Bref					
Physical	13.19	4.45	1.78	4.93	17.62
Psychological	13.11	4.08	1.41	3.92	16.32
Environmental	18.95	4.79	1.97	5.47	17.11
Life satisfaction	20.64	5.70	1.97	5.47	13.68
Impact	31.82	7.00	1.85	5.13	9.17
Total	50.59	12.26	2.74	7.60	7.92
Other PROMS					
EQ-5D-5L	0.6776	0.2492	0.1176	0.3262	32.62
MSQoL	11.566	3.601	1.6104	4.4638	18.60

%SDC is that difference as % of operational range of the scale

*SD* standard deviation, *SEM* standard error of measurement, *SDC* smallest detectable change

### Discrimination across disease subtype

While all one-way ANOVA’s showed a significance level of < 0.001 for discrimination of each domain across subtype, the non-discriminating pairwise results from the post hoc Bonferroni provide some insight (Table 4). Generally, the significant difference was driven by the contrast between Relapsing Remitting, and other subtypes. None of the domains showed a post hoc significant difference between Primary and Secondary Progressive subtypes. The only post hoc significance on the psychological domain was between Primary Progressive and Relapsing Remitting, yet the effect size of this difference was just 0.23. For the physical domain, the effect size for the difference between Primary Progressive and Relapsing Remitting was 0.54.

**Table 4:** Discrimination across disease subtype

Domain	Primary progressive A	Rapidly evolving B	Relapsing remitting C	Secondary progressive D	All cases	Scale range	Bonferroni not significant
Physical	11.9 (3.7)	13.1 (4.7)	14.2 (4.7)	11.2 (3.0)	13.2 (4.4)	0–28	A&D
Psychological	12.6 (3.8)	13.1 (4.2)	13.5 (4.1)	12.3 (3.8)	13.1 (4.1)	0–24	A&B; A&D; B&C; B&D
Environmental	17.9 (4.1)	18.3 (4.6)	19.7 (5.0)	17.6 (4.0)	19.0 (4.8)	0–32	A&B; A&D; B&D
Impact	30.3 (6.0)	31.1 (7.0)	33.1 (7.3)	29.4 (5.5)	31.8 (7.0)	0–56	A&D
Life satisfaction	19.0 (4.7)	20.7(5.5)	21.7 (6.1)	18.6 (4.4)	20.6 (5.8)	0–40	A&D; B&C
Total (24 item)	47.3 (10.3)	49.9 (11.9)	53.0 (12.9)	46.1 (9.3)	50.6 (12.2)	0–96	A&B; A&D

Mean and standard deviation (SD) on Rasch transformed measures

### Scale correlations

Correlations between the derived scales and existing subscales included in the TONiC study are shown in Table 5, all correlations were found to be significant with a *p*-value of < 0.001.

**Table 5:** Spearman’s Rho correlation coefficients between QoL measures in TONiC Study

	QoL VAS	EQ5D	Leeds QoL	WHOQOl-BREF Total	WHOQOl-BREF Impact
EQ5D	0.561				
Leeds QoL	0.520	0.537			
WHOQOl-Bref total	0.634	0.710	0.739		
WHOQOl-Bref impact	0.622	0.698	0.691	0.957	
WHOQOl-Bref life satisfaction	0.579	0.635	0.714	0.928	0.789

## Discussion

The study population included a wide range of ages and disease subtypes but with a higher proportion of patients with early and relapsing forms of MS compared with the general population. Since the study aimed to validate the performance of the WHOQOL-BREF as a measure of QOL rather than describing the absolute levels of QOL, the question of whether the subject characteristics are proportional to the overall MS population is not crucial. The study population covered the full spectrum of MS, with a large sample size and a lack of significant DIF seen in the scale performance. This demonstrates that the WHOQOL-BREF can be used in cross sectional studies to compare QOL across a broad range of MS subtypes and can be utilised in longitudinal study designs to track meaningful changes over a prolonged period of time.

It was not possible to achieve a satisfactory Rasch-based solution for the item-based analysis of the WHOQOL-BREF. Each domain showed misfit to the Rasch model requirements, multidimensionality (not the social domain) and significant DIF. As an existing scale, the super-item approach reflects how the scale is used in everyday practice, as a domain score. Allocating alternative items to the two super items imposes no a priori decisions about a domain’s structure, and there is no reason to suppose that these should reflect anything other than two identical aspects of the total score. The current analysis supports this in that most analyses showed a latent correlation of 1 between the two super-item estimates. Furthermore, the investigation of ‘substantive’ DIF has shown that, in the current study, the DIF was driven by the sample size and that, in practice, its impact on person estimates was negligible.

Given this, raw scores as a sufficient statistic for the physical, psychological and environmental scales were achieved, together with two different perspectives on the item set, namely impact and satisfaction scales, as well as a 24-item total score. Transformation from ordinal raw scores to interval scaled latent estimates were thus available but can only be used when data are complete. The social scale should be excluded as its reliability was too low for any useful application, although its three items are included in the impact/satisfaction/24-item solutions.

The precision of the original domains, as expressed by the SEM, were similar to those recently found in an observational study in oncology [33]⁠. What the current study suggests is that considering the alternative ‘impact’ and ‘life satisfaction’ domains, as well as the total score based upon all 24 items, may be more efficient, in that the percentage of the operational range of the scale to be covered to be clear of error, is smaller for those domains than the original domains. Future work to determine the minimal clinically important difference will further refine the clinical utility and interpretation of these scores.

The study included patients of varying age, sex, gender and disability representative of a broad range of patients with MS. The lack of substantive DIF suggests that the WHOQOL-BREF can be used as a generic measure of QoL in the MS population. Previous attempts to investigate whether the WHOQOL-BREF can be used as a single unidimensional construct have differed significantly in the modifications required to the scale in order to fit the Rasch model. Wang et al. required the deletion of 8 items to achieve fit to the scale due to substantial levels of DIF, thereby detracting from the reliability and original internal construct validity of the scale [34]⁠. Noerholm et al. found evidence of significant multidimensionality when applying the Danish version of the scale to the general population [35]⁠. More recently, a study used testlets to overcome the problems of local item dependency in order to demonstrate fit to the Rasch model in a UK population of patients with post polio syndrome [36]⁠. The success of the same approach (post hoc super items) in the current study provides empirical evidence to support the use of the WHOQOL-BREF as it was intended, as a universal measure of QoL that can be utilised across different diseases.

Application of Rasch analysis demonstrated the validity of these domains with a solution that did not require any alterations to the administration of the scale. The internal construct validity of each new scale can be assessed by examining the domains from which each item is derived. The impact scale contains 4 items from the physical domain and 5 each from psychological and environment. The satisfaction scale contains one item from psychological domain and 3 each from physical, social and environment. Therefore, each can be broadly thought of as an even mix of the different aspects of QoL assessed by the WHOQOL-BREF.

Whilst the WHOQOL-BREF has previously been applied in varied MS populations in observational and interventional studies and to validate novel measures [37–39]⁠, to our knowledge this is the first study to apply modern methods to investigate the psychometric properties of the scale in a population with MS. In addition, the study has provided a novel concept for using the scale. The stem of the questions in the WHOQOL-BREF suggests 2 sets of items assessing impact and satisfaction with QoL. Indeed, Hathorne and colleagues explicitly stated that the WHOQoL-BREF could be regarded as a ‘life satisfaction’ scale, given 10 of the 24 summated items focused upon satisfaction [40] (REF). Perhaps most importantly, the perspective of the scale and its individual items are those of appraisal rather than undertaking of a given task. For example, ‘How satisfied are you with the support you get from your friends?’; ‘How satisfied are you with yourself?’’; ‘How satisfied are you with your sleep?’ ‘How safe do you feel in your daily life?’ As such the WHOQoL-BREF provides a different perspective of QOL to most of those used previously for MS and can offer a useful end-point for conceptual models such as that proposed by Wilson and Cleary [6].

All correlations between measures were significant but correlations were lower between the WHOQOL-BREF and the EQ5D, which was designed as a measure of health status. Life satisfaction is an essential component of subjective well-being, thereby distinguishing the concept from health status and functioning [41]. The correlation between the new satisfaction scale and the disease-specific LMSQol included in the TONiC study found a common variance of 50%, which can be accounted for by both the appraisal perspective and the fact that the satisfaction scale addresses wider issues such as the environment and access to health care. In an RCT, the LMSQoL may be more appropriate as the impact of the specific disease is key and other social and environmental factors should be randomized out. In observational studies and where the full biopsychosocial model is applied (such as the Wilson and Cleary model), then the life satisfaction scale may be more suitable. However, the LMSQoL is less efficient than the WHOQOL-BREF as analysis of the SDD indicates it requires a change in 18.6% of the scale width to overcome random error compared with 14 and 9% of the satisfaction and impact domains, respectively, and 8% for the total score. The total score of the WHOQOL-BREF may be suitable for comparison across different diseases and populations due to its comprehensive coverage of aspects of QoL and the broad context of its development and validation. It offers a unique appraisal perspective of the lived experience of those with MS. Together with the interval scaled latent estimates derived from the Rasch model, it provides a powerful tool for use with structural equational modelling and similar methods to investigate the factors which influence perceived QoL in MS.
